# Bacteraemia, antimicrobial susceptibility and treatment among *Campylobacter*-associated hospitalisations in the Australian Capital Territory: a review

**DOI:** 10.1186/s12879-021-06558-x

**Published:** 2021-08-21

**Authors:** Cameron R. M. Moffatt, Karina J. Kennedy, Ben O’Neill, Linda Selvey, Martyn D. Kirk

**Affiliations:** 1 National Centre for Epidemiology and Population Health, Research School of Population Health, Australian National University, 2602 Canberra, ACT Australia; 2 Department of Microbiology, Canberra Hospital and Health Services, Canberra, ACT Australia; 3 School of Public Health, University of Queensland, Brisbane, QLD Australia

**Keywords:** *Campylobacter* infections, Hospitalisation, Bacteraemia, Incidence, Antimicrobial susceptibility, Antimicrobial therapy, Comorbidity, Elderly

## Abstract

**Background:**

*Campylobacter* spp. cause mostly self-limiting enterocolitis, although a significant proportion of cases require hospitalisation highlighting potential for severe disease. Among people admitted, blood culture specimens are frequently collected and antibiotic treatment is initiated. We sought to understand clinical and host factors associated with bacteraemia, antibiotic treatment and isolate non-susceptibility among *Campylobacter*-associated hospitalisations.

**Methods:**

Using linked hospital microbiology and administrative data we identified and reviewed *Campylobacter-*associated hospitalisations between 2004 and 2013. We calculated population-level incidence for *Campylobacter* bacteraemia and used logistic regression to examine factors associated with bacteraemia, antibiotic treatment and isolate non-susceptibility among *Campylobacter*-associated hospitalisations.

**Results:**

Among 685 *Campylobacter*-associated hospitalisations, we identified 25 admissions for bacteraemia, an estimated incidence of 0.71 cases per 100,000 population per year. Around half of hospitalisations (333/685) had blood culturing performed. Factors associated with bacteraemia included underlying liver disease (aOR 48.89, 95% CI 7.03–340.22, *p* < 0.001), Haematology unit admission (aOR 14.67, 95% CI 2.99–72.07, *p* = 0.001) and age 70–79 years (aOR 4.93, 95% CI 1.57–15.49). Approximately one-third (219/685) of admissions received antibiotics with treatment rates increasing significantly over time (*p* < 0.05). Factors associated with antibiotic treatment included Gastroenterology unit admission (aOR 3.75, 95% CI 1.95–7.20, *p* < 0.001), having blood cultures taken (aOR 2.76, 95% CI 1.79–4.26, *p* < 0.001) and age 40–49 years (aOR 2.34, 95% CI 1.14–4.79, *p* = 0.02). Non-susceptibility of isolates to standard antimicrobials increased significantly over time (*p* = 0.01) and was associated with overseas travel (aOR 11.80 95% CI 3.18–43.83, *p* < 0.001) and negatively associated with tachycardia (aOR 0.48, 95%CI 0.26–0.88, *p* = 0.02), suggesting a healthy traveller effect.

**Conclusions:**

*Campylobacter* infections result in considerable hospital burden. Among those admitted to hospital, an interplay of factors involving clinical presentation, presence of underlying comorbidities, complications and increasing age influence how a case is investigated and managed.

## Background

*Campylobacter* spp. are internationally significant as a cause of infectious diarrhoeal disease [1]. Most cases of infectious diarrhoea, including those caused by *Campylobacter* spp., are self-limiting, with management focused on maintenance of hydration via fluid repletion [2]. However, for persons hospitalised with *Campylobacter* infection, clinical thresholds for exclusion of bacteraemia and the consideration of antimicrobial therapy differ due to symptom severity, risk of complications or the exacerbation of underlying co-morbidities [2].

While bacteraemia is an uncommon complication of campylobacteriosis, testing and diagnosis typically occurs when a person is hospitalised [3]. Within a hospital setting, clinical tolerances and the challenge of predicting bacteraemia among febrile admissions with an enteric focus are important considerations [4]. Further, the likelihood of clinicians commencing antibiotic therapy may also increase among hospitalised cases, highlighting the importance of judicial prescribing and understanding of isolate susceptibility patterns to ensure viable treatment options remain [5].

We describe rates of bacteraemia, antimicrobial susceptibility and treatment among a cohort of *Campylobacter*-associated hospitalisations. We also examined clinical and host factors associated with the diagnosis of blood stream infections (BSI), isolate non-susceptibility and antibiotic treatment of hospitalised cases.

## Methods

### Background and data

This study forms part of a retrospective review of *Campylobacter*-associated hospital admissions in the Australian Capital Territory (ACT) between January 2004 and December 2013. A *Campylobacter*-associated hospital admission was defined as any episode of care for an admitted patient that was clinically and temporally linked to a *Campylobacter* isolate derived from the same patient. Full details of the setting, data sources, data collection and linkage are described elsewhere [6]. In summary, they include hospital generated admission data, hospital microbiology data and clinical data obtained via individual medical record review. Hospital admission details were obtained via a data extract that included patient demographics, admission and discharge dates and admission unit details. Record inclusion was determined by an inpatient admission having been assigned an International Classification of Diseases (ICD) diagnosis code ‘A045—*Campylobacter* enteritis’. We also obtained a separate microbiology data extract detailing inpatient isolations of *Campylobacter* spp., including specimen type, collection dates and antimicrobial susceptibility data. All laboratory diagnoses of *Campylobacter* infection were made via culture; speciation was not routinely performed on hospital isolates prior to 2013. Susceptibilities to ciprofloxacin, nalidixic acid and erythromycin were assessed using disk diffusion and according to Clinical and Laboratory Standards Institute breakpoints [7]. Intermediate and resistant isolates were grouped as non-susceptible to aid analysis. Due to the potential for multiple specimens being collected during an individual admission (or across multiple related admissions), we reported antimicrobial susceptibility using the earliest available data. Microbiology results were then linked to both admissions with and without ICD code ‘A045’. We undertook a review of medical records to collect additional details on illness presentation, associated complications, patient co-morbidities (as per the Charlson Co-morbidity Index) [8] and antibiotic treatment and prescribing details (e.g., antibiotic type, dosage, frequency and administration route). For antibiotic treatment, we defined a total daily dose as the dosage in milligrams (mg) multiplied by the frequency of administration per day, with the product expressed in milligrams per day.

### Statistical analysis

We calculated bacteraemia incidence per 100,000 persons using the ACT’s mid-year estimated resident population for each year between 2004 and 2013 [9]. We used non-parametric methods, including median tests, to analyse non-normally distributed variables such as age. We used Pearson’s chi-squared test and Fisher’s exact tests to assess simple statistical associations between key outcome and independent variables of interest, while trends in proportions were assessed using chi-squared tests. Preliminary analyses included the estimation of relative risks (RRs), 95% confidence intervals (CIs) and p-values to assess potential predictors of blood culturing, antimicrobial susceptibility and antibiotic treatment. Variables examined included age, sex, country of birth, previous *Campylobacter*-associated hospitalisation, overseas travel history, signs and symptoms, admission unit, comorbidities and the presence of key signs of infection. Using a stepwise additive approach, with the most significant variables being added first, we constructed separate logistic regression models to identify predictors associated with (i) collection of blood specimens for culture, (ii) positive blood isolates, (iii) non-susceptibility to ciprofloxacin, (iv) any antimicrobial non-susceptibility and (vii) antibiotic treatment during hospitalisation. Erythromycin non-susceptibility was not assessed due to the limited number of observations. Clinical relevance and statistical evidence were used to assist with variable selection for multivariable analysis. The significance level for removal from the models was set at p ≤ 0.05. We used likelihood-ratio tests to assess the explanatory power of the models, with the variable expressing the largest p-value being removed. Final results were expressed as adjusted odds ratios (aOR), with accompanying 95% confidence intervals and *p*-values. We used Hosmer-Lemeshow tests to assess the goodness-of-fit for each model. All statistical analyses were performed using Stata v.14 (StataCorp, USA).

## Results

### *Campylobacter* bacteraemia

**Fig. 1:**
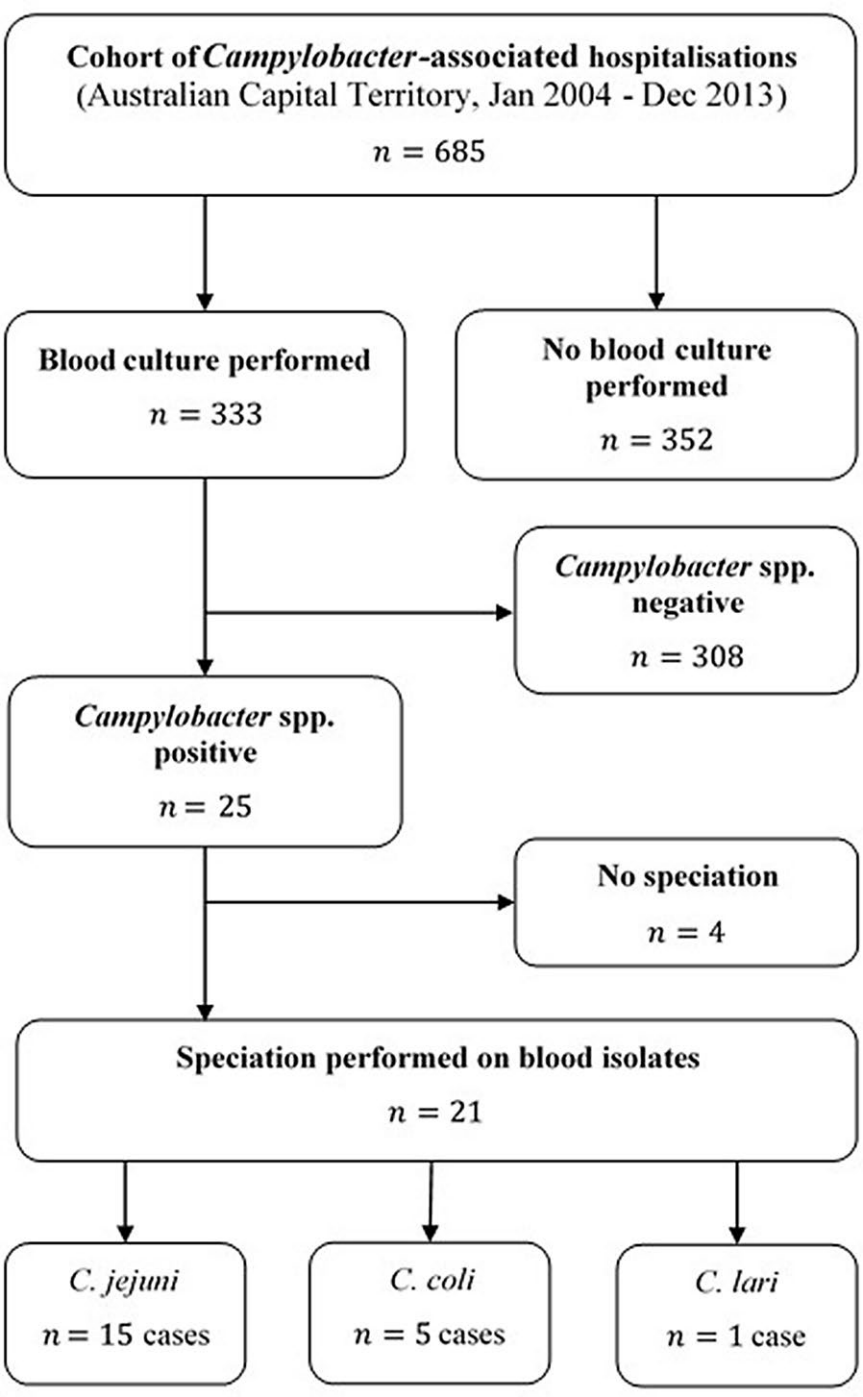
Flow diagram for blood culture collection and subsequent *Campylobacter* spp. bacteraemia among a cohort of Campylobacter-associated hospitalisations

**Fig. 2:**
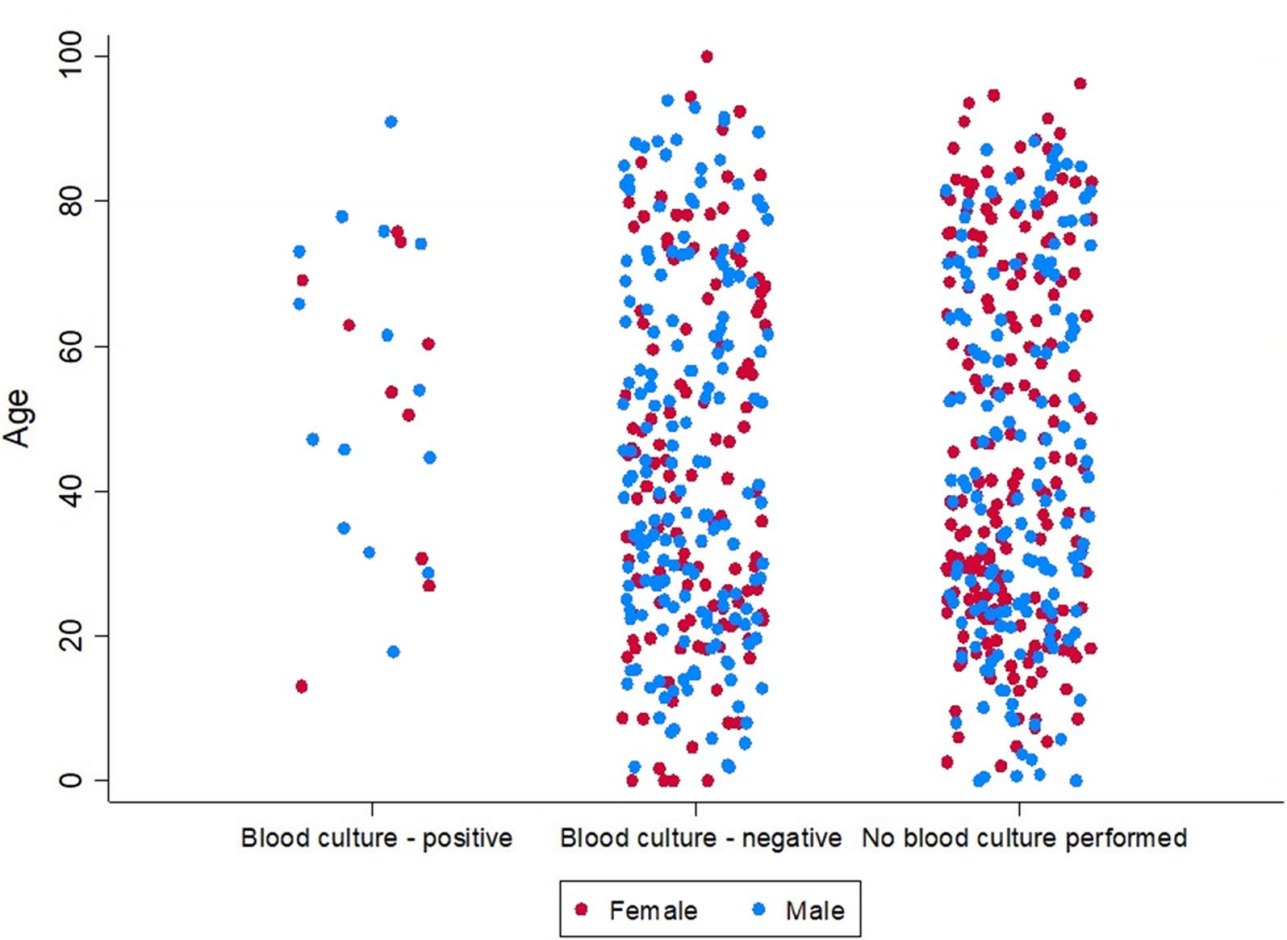
Jitter plot of age and sex by blood culture status among *Campylobacter*-associated hospitalisations

Out of 685 admissions, 333 (49%) had blood drawn for culture and 25 (7.5%) of these tested positive (Fig. 1**)**. Speciation was performed on 21 (84%) of these case isolates with 15 (71.4%) cases of *C. jejuni*, 5 (23.8%) cases of *C. coli* and a single (4.0%) case of *C. lari* bacteraemia. Amongst the 25 admissions with bacteraemia, 15 were male (60%) and 10 were female (40%), while the median age was 59.5 years (range 12 to 90 years). Amongst 308 negative cases there were 177 males (57.5%) and 131 females (42.5%), with a median age of 38.8 years (age range $<1.0$ to 92 years). This age difference was significant (median test${ \chi }^{2}=5.30$, $p=0.02$) but no evidence of a difference in the sex composition was observed (${\chi }^{2}=0.06$, $p=0.81$). The age and sex of admissions in relation to blood culture status is shown in Fig. 2. We also compared those who had blood drawn for culture (whether positive or negative) with those that did not to assess demographic differences. We saw no evidence of a difference in median age (median test${\chi }^{2}=0.18$, $p=0.68$) but did observe males to be more likely to have blood drawn (${\chi }^{2}=11.17$, $p=0.001$). We observed no difference in the proportion of admissions with documented comorbidities who had blood specimens collected for culture when compared to admission without comorbidities who had blood taken for culture ${(\chi }^{2}=1.42$, $p=0.23$). Bacteraemia generally occurred in the context of antecedent diarrhoeal illness, although two admissions involved primary bacteraemia (i.e. without diarrhoea) in patients with haematological malignancies. Comorbidities, while common, were not a characteristic feature of cases hospitalised with bacteraemia as shown in Table 1. No deaths were identified among cases with bacteraemia.

**Table 1:** Characteristics of cases hospitalised with *Campylobacter* bacteraemia, 2004 to 2013

Year (case)	Age range/sex	Species	Bacteraemia—source	Antimicrobial susceptibility	Antimicrobial treatment	Significant medical history and risk factors
2004	80 + M	*C. jejuni*	Enteric—secondary	Fully sensitive	Nil	Age, nil other significant
2005 (a)	50–59 F	*Campylobacter* sp.	Enteric—secondary	Fully sensitive	PO ciprofloxacin 500 mg bd	Acute myeloid leukaemia
2005 (b)	60–69 F	*Campylobacter* sp.	Enteric—secondary	Fully sensitive	Nil	Nil significant
2005 (c)	40–49 F	*C. jejuni*	Enteric—secondary	Fully sensitive	PO ciprofloxacin 500 mg bd	IV drug use, PUD on omeprazole
2006 (a)	40–49 M	*C. jejuni*	Enteric— secondary	Fully sensitive	PO ciprofloxacin 500 mg bd	Untreated Stage III HIV
2008 (a)	20–29 M	*Campylobacter* sp.	Enteric—secondary	Fully sensitive	IV azithromycin 500 mg qd	Nil significant
2008 (b)	60–69 M	*C. coli*	Primary bacteraemia	Ciprofloxacin- Resistant Erythromycin- Resistant	Nil	Lymphocytic lymphoma
2009	30–39 F	*C. jejuni*	Enteric—secondary	Fully sensitive	PO ciprofloxacin 250 mg bd	History of renal transplant secondary to IgA nephropathy
2010 (a)	60–69 M	*Campylobacter* sp.	Enteric—secondary	Fully sensitive	PO ciprofloxacin 500 mg bd	Bowel carcinoma, current chemotherapy
2010 (b)	30–39 M	*C. coli*	Enteric—secondary	Fully sensitive	PO ciprofloxacin 500 mg bd	Alcoholic liver disease with portal hypotension and bleeding varices
2010 (c)	70–79 F	*C. jejuni*	Enteric—secondary	Fully sensitive	PO ciprofloxacin 500 mg bd	T2DM
2010 (d)	10–19 F	*C. jejuni*	Enteric—secondary	Fully sensitive	PO azithromycin 500 mg qd (upon discharge)	Nil significant.
2010 (e)	70–79 F	*C. coli*	Enteric—secondary	Fully sensitive	PO ciprofloxacin 500 mg bd	T2DM
2010 (f)	40–49 M	*C. jejuni*	Enteric—secondary	Fully sensitive	PO Norfloxacin 400 mg bd	Irritable bowel syndrome
2011 (a)	60–69 M	*C. lari*	Enteric—secondary	Ciprofloxacin— Resistant	PO Doxycycline 100 mg bd	Alcoholic liver disease with portal hypotension, recent intracerebral bleed
2011 (b)	80 + M	*C. jejuni*	Enteric—secondary	Fully sensitive	Nil prescribed	Age, nil other significant
2011 (c)	70–79 M	*C. coli*	Enteric—secondary	Fully sensitive	PO ciprofloxacin 500 mg bd	Asplenic
2012 (a)	40–49 M	*C. jejuni*	Enteric—secondary	Fully sensitive	IV ciprofloxacin 500 mg bd	Multiple sclerosis, current chemotherapy pre-stem cell transplantation, IDDM
2012 (b)	30–39 F	*C. jejuni*	Enteric—secondary	Fully sensitive	Nil	Pregnant 33/40K, IDDM
2012 (c)	70–79 M	*C. jejuni*	Enteric—secondary	Ciprofloxacin— Resistant Nalidixic acid— Resistant	Nil	Diabetic neuropathy, chronic renal failure
2012 (d)	60–69 F	*C. coli*	Enteric—secondary	Ciprofloxacin— Resistant Nalidixic acid— Resistant	Nil	Nil significant
2013 (a)	20–29 M	*C. jejuni*	Enteric—secondary	Fully sensitive	Nil	Nil significant
2013 (b)	20–29 M	*C. jejuni*	Primary bacteraemia	Fully sensitive	PO Ciprofloxacin 750 mg bd (upon discharge)	B cell leukaemia, AVN (on steroids), SIADH
2013 (c)	50–59 M	*C. jejuni*	Enteric—secondary	Fully sensitive	PO ciprofloxacin 500 mg bd	Liver failure with cirrhosis, portal hypotension secondary to Hepatitis C, T2DM, hypothyroidism. Awaiting transplant.
2013 (d)	70–79 M	*C. jejuni*	Enteric—secondary	Fully sensitive	Nil	Age, nil other significant

PO, per oral; IV ,  intravenous; PUD, peptic ulcer disease; HIV, human immunodeficiency virus; T2DM, type 2 diabetes mellitus; IDDM, insulin dependent diabetes mellitus; AVN, acute vascular necrosis; SIADH, syndrome of inappropriate antidiuretic hormone

**Fig. 3:**
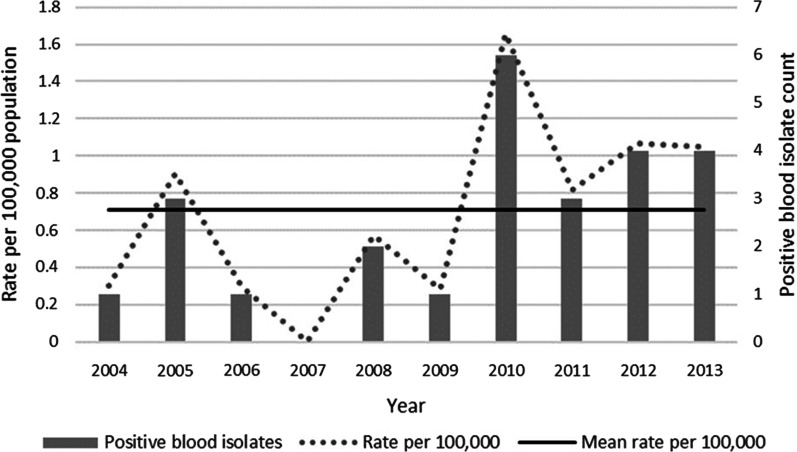
Incidence of *Campylobacter* spp. bacteraemia among ACT residents, 2004 to 2013

During the period 2004 to 2013, the mean incidence of *Campylobacter* bacteraemia in the host population was 0.71 cases per 100,000 population per year (95% CI 0.48–1.05 per 100,000 population) (Fig. 3). We did not observe temporal trends in the proportion of *Campylobacter*-associated hospitalisations undergoing blood collection for culture or in the proportion of positive blood cultures among *Campylobacter*-associated hospitalisations.

Factors associated with the collection of blood samples for culture and subsequent isolation of *Campylobacter* spp. are shown in Tables 2 and 3.

**Table 2:** Factors associated with collection of blood for culture among a cohort of *Campylobacter*-associated hospitalisations ($\varvec{n}=663$)

Predictor variable	Estimated adjusted Odds Ratio (aOR)	95% Confidence interval	$\varvec{p}$ -value
Infectious Diseases Unit admission	8.00	1.58–40.61	0.01
Febrile during admission (≥ 38 °C)	21.30	13.76–32. 96	< 0.001
Tachycardia	1.75	1.13–2.72	0.01
Moderate to severe renal disease	2.87	1.26–6.55	0.01
10–19 years age group	0.36	0.19–0.71	< 0.01

22 observations contained missing data

Hosmer and Lemeshow goodness of fit ${\chi }^{2}=2.70$, $p=0.61$

**Table 3:** Factors associated with blood stream isolation of *Campylobacter* spp. among *Campylobacter-*associated hospitalisations (*n* = 333)

Predictor variable	Estimated adjusted Odds Ratio (aOR)	95 % Confidence interval	$\text{p}$ -value
Moderate to severe liver disease	48.89	7.03–340.22	< 0.001
Haematology Unit admission	14.67	2.99–72.07	0.001
Age group 70–79 years	4.93	1.57–15.49	< 0.01
Admission during summer months	2.93	1.14–7.57	0.03
Indigenous Australian	10.87	1.00–117.89	0.05

Hosmer and Lemshow goodness of fit ${\chi }^{2}=0.71$, $p=0.70$

### Antimicrobial susceptibility

We identified 548 *Campylobacter*-associated admissions with a primary isolation of *Campylobacter* spp. and where subsequent antimicrobial susceptibility testing was performed. For 526 (96%) of these cases there was available data for three standard antimicrobials, ciprofloxacin, nalidixic acid and erythromycin. Of the remainder, nalidixic acid data was unavailable for 21 isolates, with erythromycin susceptibility unavailable for a single isolate.

**Fig. 4:**
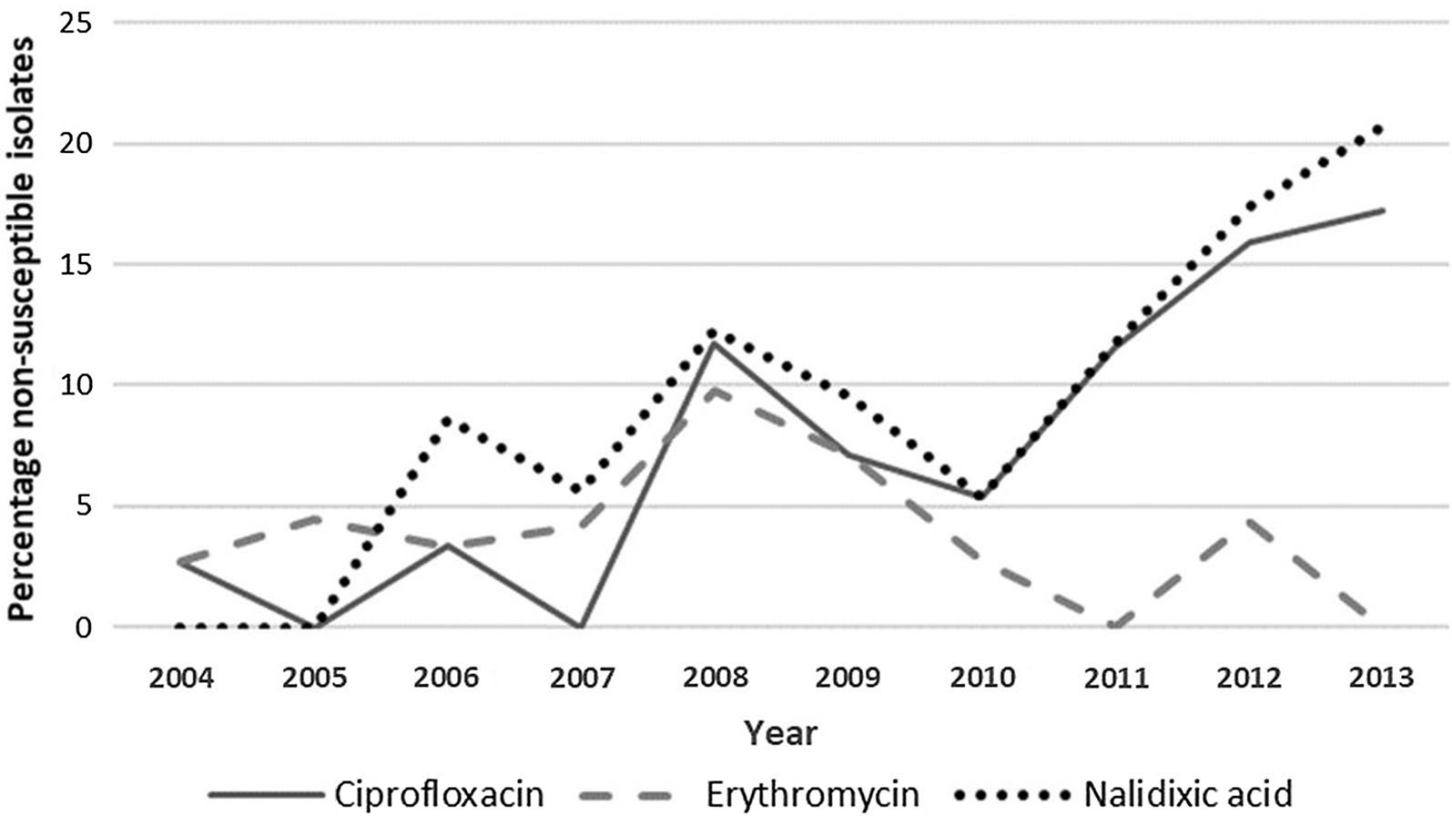
Non-susceptibility to standard antimicrobials from *Campylobacter* isolates obtained during *Campylobacter*-associated hospitalisations, 2004 to 2013

During the study period, 13% (70/548) of primary isolates exhibited non-susceptibility to at least one standard antimicrobial. The proportion of non-susceptible isolates ranged from ≤ 5.0% in 2004/05 to > 20.0% in 2012/13, with this increase being significant (${\chi }^{2}=6.12$, $p=0.01$) (Fig. 4). Among the individual antimicrobials, nalidixic acid non-susceptibility was reported for 9% (49/527) of tested isolates, 7% (40/548) for ciprofloxacin and 4% (21/547) for erythromycin.

Figure 4 shows that lower rates of erythromycin non-susceptibility were observed while significant increases in both ciprofloxacin (χ^2^ = 16.51, *p* < 0.001) and nalidixic acid non-susceptibility occurred during the study period (χ^2^ = 10.85, *p* = 0.001). Factors associated with antimicrobial non-susceptibility among *Campylobacter*-associated hospitalisations are shown in Tables 4 and 5.

**Table 4:** Factors associated with ciprofloxacin non-susceptible isolates among *Campylobacter*-associated hospitalisations (*n* = 411)

Predictor variable	Estimated adjusted Odds Ratio (aOR)	95 % Confidence interval	*p*-value
Bloody diarrhoea	0.30	0.09–1.03	0.06
Recent overseas travel	15.53	2.86–84.18	0.001
Previous *Campylobacter*-associated hospitalisation	12.87	1.72–96.12	0.01

Hosmer and Lemeshow goodness of fit χ^2^  = 0.03,  *p* = 0.86

**Table 5:** Factors associated with non-susceptibility to ciprofloxacin, nalidixic acid or erythromycin among a cohort of *Campylobacter*-associated hospitalisations (*n* = 548)

Predictor variable	Estimated adjusted Odds Ratio (aOR)	95 % Confidence Interval	*p*-value
Recent overseas travel	11.80	3.18–43.83	< 0.001
Previous *Campylobacter*-associated hospitalisation	17.09	2.65−110.07	< 0.01
Tachycardia	0.48	0.26–0.89	0.02

Hosmer and Lemeshow goodness of fit χ^2^  = 0.01,  *p* = 0.92

### Antibiotic treatment

Antimicrobial treatment was provided for 32% (219/685) of *Campylobacter*-associated hospitalisations. Those receiving treatment were observed to be significantly older (median 48.5 years, range 8 years to 92 years) than admissions where antibiotics were not administered (median 34.8 years, range < 1 years to 91 years, χ^2^ = 26.92, *p* < 0.001). No sex-based differences were observed. Treatment associated with bacteraemia is described in Table 1.

Second generation fluoroquinolones were used in 79% (172/219) of treated admissions. Ciprofloxacin was administered to 82.0% (141/172), with the remainder receiving norfloxacin. Those receiving ciprofloxacin were younger (median 47.2 years, minimum 12 years, maximum 90 years) compared with those receiving norfloxacin (median age 64.3 years, minimum 19 years to maximum 92 years), but this difference was not significant. For admissions treated with ciprofloxacin, 91.4% (129/141) received treatment orally, with a median total daily dose of 1000 mg. For those receiving parenteral ciprofloxacin ($n$ =12), the median total daily dose was 800 mg. The median total daily dosage for oral norfloxacin ($n$ =31) was 800 mg.

Macrolides (azithromycin or erythromycin) were administered to 21.0% (46/219) of admissions, with 63.0% (29/46) receiving azithromycin. No age or sex differences were observed between those receiving azithromycin versus erythromycin. For admissions receiving azithromycin, 72.4% (21/29) received treatment orally, with a median total daily dose for both oral and IV azithromycin of 500 mg. Oral administration was provided for 94.1% (16/17) of admissions receiving erythromycin, with a median total daily dose of 2000 mg. One patient received an initial total daily dose of 3200 mg IV erythromycin, prior to this being reduced to a total daily dose of 1600 mg. For two admissions (involving the same patient), a tetracycline (doxycycline) was used to treat a *C. lari* bacteraemia and enterocolitis.

A significant increase in the proportion of *Campylobacter*-associated hospitalisations being administered antimicrobials was observed over time (χ^2^ = 4.37, *p* = 0.04), rising from 27% (14/52) in 2004 to 38% (26/68) of admissions in 2013. No difference in the proportion of admissions treated with either fluoroquinolones or macrolides was observed. Among admissions receiving macrolides a significant increase in the administration of azithromycin over erythromycin was observed (χ^2^ = 16.31, *p* < 0.001), most notably from 2011 onwards. No changes over time in the proportion of admissions treated with ciprofloxacin versus norfloxacin were observed. Factors associated with administration of antibiotics during *Campylobacter*-associated hospitalisations are shown in Table 6.

**Table 6:** Factors associated with antibiotic administration among a cohort of *Campylobacter*-associated hospitalisations (*n* = 607)

Predictor variable	Estimated adjusted Odds Ratio (aOR)	95 % Confidence Interval	*p*-value
Age 0–9 years	0.07	0.01–0.57	0.01
Age 10–19 years	0.44	0.20–0.97	0.04
Age 40–49 years	2.34	1.14–4.79	0.02
Emergency Unit admission	0.06	0.02–0.17	< 0.001
Gastroenterology admission	3.75	1.95–7.20	< 0.001
Gen. Medicine admission	2.02	1.22–3.35	< 0.01
Infectious Diseases admission	2.58	1.03–6.44	0.04
Vomiting	1.70	1.11–2.61	0.02
Electrolyte imbalance	1.73	1.12–2.67	0.01
Blood specimen for culture	2.76	1.79–4.26	< 0.001

Hosmer and Lemeshow goodness of fit χ^2^ 9.23,  *p* = 0.32

## Discussion

We observed a high rate of bacteraemia in this study of *Campylobacter*-associated hospital admissions. Although blood cultures are not routinely performed for cases admitted with infectious gastroenteritis, we observed for *Campylobacter*-associated hospitalisations the presence of fever, pre-existing kidney disease or admission to particular subspecialty units increased the likelihood of blood being collected. Comorbidity and advanced age were both associated with a subsequent isolation from blood. One third of admissions received antibiotic treatment during the study period, with the proportion of those treated rising significantly over time. We also observed a temporal increase in the proportion of isolates exhibiting non-susceptibility to standard antimicrobials, notably fluoroquinolones. This is notable given the clinical and public health concern about development of antimicrobial resistance. Factors associated with ciprofloxacin resistance included recent overseas travel and previous hospitalisation for campylobacteriosis. This carries important clinical consequences, as the option to treat with antibiotics may carry greater importance among hospitalised cases compared to non-hospitalised cases.

Campylobacteriosis most commonly presents as a self-limiting enterocolitis, with secondary bacteraemia a recognised, but relatively infrequent complication [10]. A number of studies have sought to determine the incidence of *Campylobacter* bacteraemia in high-income settings, estimating rates to be between 0.20 and 0.47 cases per 100,000 population [3, 11, 12]. A more recent Swedish study [13] has reported an incidence of 1.00 case per 100,000 population, with this linked to changes in automated blood culture collection systems. In our study we observed only 25 incident cases of bacteraemia, equating to a mean incidence of 0.71 cases per 100,000 population. While bacteraemia is relatively infrequent, our population rates appears high, likely reflecting high background incidence of campylobacteriosis in the ACT [14] and a lower threshold for testing among hospitalised cases.

Blood cultures are the gold standard for the diagnosis of BSIs [4]. In our study, patients who had a measured fever (≥ 38 °C), underlying chronic kidney disease or who were admitted under the care of the Infectious Diseases Unit were more likely to have blood drawn for culture. Fever is a common prompt for blood cultures, with patients whose measured temperatures are ≥ 38 °C having increased likelihood of bacteraemia [15]. Similarly tachycardia, another vital sign, has also been used in clinical prediction rules for blood-stream infection [16]. Although comorbidities do not feature in BSI underlying kidney disease has also been shown as a risk factor for bloodstream infections in older patients [17]. Several consequences of kidney disease have been proposed to contribute to infection including malnutrition, chronic inflammation, retained uremic solutes, trace element deficiencies and metabolic abnormalities [18]. The finding that blood culture is more likely to be ordered by Infectious Diseases clinicians is unsurprising given the clinical focus of this subspecialty. Conversely, there was significant evidence that those aged between 10 and 19 years were less likely to have blood cultures performed. This finding likely reflects fewer hospitalisations being observed due to the low incidence of campylobacteriosis in this age grouping [14].

Predicting BSIs is challenging with numerous models developed for specific populations, settings and sources of infection [19]. The pre-test probability of bacteraemia will therefore vary considerably based upon the clinical context and source of infection [15]. Generally only 5–10% of blood cultures are positive, and of those positive results, between 30 and 50% represent contaminants [19]. In our study we observed blood cultures to be routinely requested, with 49% of acute admissions having blood drawn and 7% being positive for *Campylobacter* spp. These admissions involved both immunosuppressed and immunocompetent patients. The impacts of comorbid and immunosuppressive conditions on clinical variables used in predicting bacteraemia is less well understood [15], although associations with campylobacteriosis and invasive disease in immunosuppressed patients have been described [3, 20]. Transient bacteraemia may also be common among immunocompetent hosts with *Campylobacter* enterocolitis but is less frequently detected due to the bactericidal effect of human serum and reduced frequency of blood culture among acute enterocolitis patients [10], although nearly half of acute admissions in our study had blood culturing performed. Notably, similar proportions of acute admissions with and without comorbidities were observed to have blood drawn for culture suggesting in our study population that comorbidity exerted limited influence on decisions to request blood cultures. This is perhaps not surprising given prediction rules for BSI focus on clinical signs [15].

### Factors associated with a positive blood stream isolate (bacteraemia)

The statistical association we observed between cases with positive blood cultures and the presence of underlying liver disease and haematological malignancy is in keeping with hospital-based studies showing higher proportions of these conditions among cases with *Campylobacter* bacteraemia [11, 21, 22]. In addition, advanced age was also statistically associated with detection of bacteraemia, a characteristic seen in larger population-based studies of campylobacteriosis [3]. Several studies also report seasonality with *Campylobacter* bacteraemia [23, 24]. Our results show hospitalisation during the southern hemisphere summer to be associated with bacteraemia, a finding that aligns with the seasonality of *Campylobacter* enteritis in Australia [14]. A further association with blood culture positivity was Indigenous status. This result was derived however from a small number of observations with, Indigenous Australians comprising 1% of *Campylobacter*-associated hospitalisations in the ACT [6].

### Factors associated with non-susceptibility to antimicrobials

We observed statistically significant evidence of an increase in the proportion of isolates exhibiting non-susceptibility to standard antimicrobials, with this increase driven primarily by non-susceptibility to fluoroquinolones (including nalidixic acid). In keeping with trends in comparable settings only low rates of non-susceptibility to macrolides were observed [25, 26]. Internationally, fluoroquinolone resistance among *Campylobacter* spp. has become a major public health problem [5]. Increases in the proportion of clinical isolates demonstrating ciprofloxacin resistance has been observed in the United Kingdom (UK) and United States (US) [25, 27], while in the European Union (EU) more than half (54.6%) of human-associated *C. jejuni* and two-thirds (66.6%) of *C. coli* isolates were resistant to ciprofloxacin in 2013 [28].

Australia has previously reported low rates of fluoroquinolone non-susceptibility among clinical *Campylobacter* isolates (around 2% in 2006) [29]. This has been credited to a national pharmaceutical subsidy scheme that restricted human quinolone use and through regulation forbidding quinolone use in food-producing animals [30]. More recent studies reveal this situation has changed markedly, with current rates of ciprofloxacin resistance in clinically-derived *Campylobacter* isolates now ranging between 13 and 20% [31, 32].

Overseas travel is a well-established risk factor for the acquisition of ciprofloxacin-resistant *Campylobacter* infections [26, 33]. In our study, cases with ciprofloxacin resistance all reported travel to India and South-East Asia, destinations associated with high rates of antimicrobial resistance among enteric pathogens (including *Campylobacter* spp.) [34, 35]. Nevertheless, the majority of ciprofloxacin non-susceptible isolates in our study had no recent overseas travel identified, meaning factors associated with domestic acquisition of ciprofloxacin resistance require greater consideration. US data similarly shows increases in domestically acquired ciprofloxacin resistance [27].

We observed that ciprofloxacin non-susceptibility was also associated with previous hospitalisation with campylobacteriosis. There is a paucity of population-level data on recurrent hospitalisations involving non-susceptible *Campylobacter* isolates. However, rates of recurrent campylobacteriosis in community settings have been reported to be as high as 248 episodes per 100,000 cases per year in the five years following an initial infection [36]. Explanations for our finding could be either host or pathogen-related, including higher rates of humoral immunodeficiency in patients hospitalised with recurrent campylobacteriosis [11, 37] or because of *de novo* mutations or increases in resistant organisms already present at subclinical levels.

While there has been debate around isolate non-susceptibility and disease severity, reanalysis of the issue appears to show no substantial clinical differences between resistant and susceptible *Campylobacter* isolates [38]. Consequently, our finding of reduced odds for bloody diarrhoea and tachycardia among admissions with ciprofloxacin non-susceptibility and any antimicrobial non-susceptibility respectively, most likely represents the so-called “healthy traveller” effect [39].

### Factors associated with antibiotic treatment during admission

During the study period we observed statistically significant evidence of an increase in the proportion of *Campylobacter*-associated hospitalisations receiving antibiotic treatment. Antimicrobial therapy for *Campylobacter* enterocolitis is not routinely advised but may be recommended for patients with or at risk of severe disease, including high volume or bloody diarrhoea, high fever, symptom duration greater than one week, pregnancy or immunocompromised status [2, 10, 40]. Given that hospitalisation can be viewed as a marker of disease severity [41], the rates of treatment within our study population might be expected to differ from those among non-hospitalised campylobacteriosis cases.

Other research on campylobacteriosis in the ACT has found no concomitant increase in hospitalisations during the same period as the current study [6]. Antibiotic treatment rates may be a reflection of local treatment practices rather than a response to disease severity, with data showing rates of appropriate prescribing and compliance with antibiotic guidelines in ACT hospitals to be the lowest in Australia during the study period [42].

One-third of *Campylobacter*-associated hospitalisations received antibiotics, either empirically or as targeted therapy for confirmed campylobacteriosis. Second generation fluoroquinolones—mainly per oral ciprofloxacin—comprised 80% of treatment, with macrolides the remainder. Australia has been successful in efforts to limit use of quinolones in humans and to prohibit their use in food-producing animals [30]. This has preserved their clinical use in Australia, with ciprofloxacin and norfloxacin remaining as empirical treatment options for acute infectious diarrhoea, while being recommended alongside azithromycin for treatment of domestically acquired *Campylobacter* enteritis [40]. Conversely, the high rates of quinolone resistance experienced in the UK, EU and US has seen macrolide treatment recommended or a greater emphasis placed on travel history and knowledge of local resistance patterns to guide empirical prescribing [43, 44].

Within our hospitalised study population, the strongest predictor of antibiotic treatment was collection of a blood specimen for culture. Both antibiotic prescription and ordering of blood cultures are clinical decisions, suggesting that the underlying clinical context observed by treating clinicians impacts both practices inducing them to be positively associated. Admission under specific clinical units, including Gastroenterology, Infectious Diseases and General Medicine were also associated with increased odds of receiving antimicrobial therapy. Hospitalisation implies a higher level of morbidity, potentially explaining the higher likelihood of antibiotic administration. Variation in treatment focus could also be expected between subspecialties, especially when underlying comorbidities are exacerbated. Such decision making may be further influenced by routine clinical behaviour, unease regarding the consequences of BSI or by the acceptability of not obtaining blood cultures among particular specialities [4].

Age was also found to be an important factor in treatment, with paediatric admissions being less likely to receive antibiotics. This finding reflects that fluoroquinolones—the most commonly prescribed antibiotic class— are not recommended for use in children due to safety concerns [45]. We also observed that patients aged 40–49 years were more likely to be prescribed antibiotics. Reasons for this are less certain, but around 20% of admissions to high prescribing units such as Gastroenterology and Infectious Diseases were in this age range.

Vomiting and electrolyte imbalance were also associated with provision of antibiotic therapy. Vomiting is a less frequently reported symptom of campylobacteriosis but serves as an indicator for disease severity [46] and a predictor of bacteraemia [47]. While we did not assess the severity of dehydration, it is likely that a population such as ours included a higher proportion of cases with more pronounced symptomatology and severity of symptoms compared with non-hospitalised cases.

## Limitations

There are a number of potential limitations with our study. Firstly, our rates of bacteraemia may underestimate the true incidence, as we identified cases using only public hospital laboratory data. Other cases of *Campylobacter* bacteraemia may have been diagnosed and managed in the community or the private hospital sector, although the clinical significance of these is less certain. A second limitation relates to the precision of our model estimates, with the small numbers of observations for some outcomes making detection of clinically meaningful associations challenging. Despite this, the observed associations still align plausibly with *Campylobacter’s* epidemiology. A third limitation relates to the generalisation of our findings. Our study population was hospital-based and drawn from a single Australian territory, with regional and international differences in the epidemiology of campylobacteriosis being observed in high income settings [14, 22, 48]. Finally, antimicrobial susceptibility testing and bacterial speciation were not performed on all isolates, limiting exploration of species-specific features such as higher macrolide resistance rates among *C. coli* isolates [49].

## Conclusions

*Campylobacter* infections cause a substantial disease burden, as reflected by the high number of hospitalisations and high incidence of bacteraemia in our study. While a spectrum of illness can be observed among hospitalisations, many cases exhibit signs suggestive of systemic disease. Furthermore, both the proportion of cases receiving antibiotic treatment and those having isolates that were non-susceptible to standard antimicrobials increased over time. Given the increasing incidence of *Campylobacter* infections, particularly among older patients, understanding hospitalisation burden becomes increasingly important. This study provides some evidence in relation to clinical factors influencing the management of hospitalised cases in high income settings.

## Data Availability

The data that support the findings of this study are available from the ACT Government Health Directorate and Calvary Health Care (Bruce). Restrictions apply to the availability of these data, which were used under approvals for the current study and so are not publicly available. Data are however available from the authors upon reasonable request and with permission of the ACT Government Health Directorate and Calvary Health Care (Bruce).

## Abbreviations

ACT: Australian Capital Territory
aOR: Adjusted Odds Ratio
BSI: Blood Stream Infection
CI: Confidence Interval
EU: European Union
ICD: International Classification of Diseases
UK: United Kingdom
US: United States
